# College Student E-Cigarette Users’ Knowledge about E-Cigarettes: Ingredients, Health Risks, Device Modifications, and Information Sources

**DOI:** 10.3390/ijerph19041962

**Published:** 2022-02-10

**Authors:** Alison C. McLeish, Joy L. Hart, Kandi L. Walker

**Affiliations:** 1 Department of Psychological and Brain Sciences, University of Louisville, Louisville, KY 40292, USA; 2 VapeRace Center, Christina Lee Brown Envirome Institute, School of Medicine, University of Louisville, Louisville, KY 40202, USA; joy.hart@louisville.edu (J.L.H.); kandi.walker@louisville.edu (K.L.W.); 3 Tobacco Center for Regulatory Science, American Heart Association, Dallas, TX 75231, USA; 4 Department of Communication, University of Louisville, Louisville, KY 40292, USA

**Keywords:** behavior, college students, e-cigarettes, health risks, knowledge, information seeking, e-cigarette ingredients, device modification

## Abstract

Although college students represent a growing demographic of e-cigarette users, it is unclear how knowledgeable they are about the product they use. The lack of such knowledge could result in unsafe practices and greater health risks. Therefore, the purpose of the current study was to examine college student e-cigarette users’ knowledge about e-cigarette ingredients and health risks, how often they modify their devices, and whether they utilize reputable sources when searching for information regarding e-cigarettes. The participants were 183 undergraduate e-cigarette users (*M_age_* = 19.98, *SD* = 1.98; 71.6% female; 85.8% White). Most participants correctly recognized that e-cigarettes increase the risk of cardiovascular disease and lung disease, but fewer than half recognized the increased risk of seizures and depression. Only one-third to one-half of participants correctly identified the toxic compounds commonly found in e-cigarettes, and most indicated that they would consult Google or a friend with questions about e-cigarettes. College student e-cigarette users are well-informed about many health risks associated with e-cigarettes. However, they are relatively unaware of the harmful substances in e-cigarettes and are seeking information from less reliable sources. Targeted public health campaigns educating college students about e-cigarettes, including where to seek reliable information, are needed.

## 1. Introduction

Although the period of adolescence tends to receive the most attention, the rates of e-cigarette use among college students in the U.S. have increased exponentially in recent years [1]. In fact, recent rates of e-cigarette initiation are nearly twice as high among young adults compared to teens [2]. The increase in e-cigarette use has been driven, in part, by beliefs that e-cigarettes are less harmful than combustible cigarettes [3,4]. Unfortunately, mounting evidence indicates that e-cigarettes pose significant health risks, although these risks may be lower than for combustible cigarettes. There is substantial evidence of the increased risk of respiratory diseases and growing evidence of the increased risk of cardiovascular diseases [5,6,7]. Additionally, emerging evidence suggests an association between e-cigarettes and seizures in youth and young adults [8]. In addition to physical health risks, there are significant associations between e-cigarette use and depression [9,10,11].

As the evidence regarding increased physical and mental health risks associated with e-cigarette use relative to no tobacco use continues to accumulate, the degree to which college student e-cigarette users are knowledgeable about the ingredients contained in e-cigarettes, as well as the health risks associated with their use, is unclear. Studies that were carried out among adult e-cigarette users indicate that the majority cannot identify specific ingredients in e-cigarettes beyond nicotine, nor are they aware of the long-term health risks associated with their use [12,13]. However, to our knowledge, such an examination has not been conducted in college students.

Moreover, there has been little exploration of users’ behavior that might exacerbate these health risks, such as making unsafe device modifications (e.g., changing the voltage) [14]. Further, given how quickly the e-cigarette product landscape can change, it is important to understand where users go when they have questions about e-cigarettes. Therefore, the purpose of the current study was to examine college student e-cigarette users’ knowledge about e-cigarette ingredients and health risks, how often they make changes to their devices that might exacerbate risk, and what sources they utilize when searching for information regarding e-cigarettes. A secondary aim was to explore whether users’ knowledge and behaviors differed by gender, race, or year in school.

## 2. Materials and Methods

### 2.1. Participants

The participants included 183 undergraduates (*M_age_* = 19.98, *SD* = 1.98; 71.6% female) who had engaged in e-cigarette use in the past 30 days and had not engaged in combustible cigarette use in the past 30 days. In terms of racial and ethnic background, 8.2% were Hispanic in ethnicity and 85.8% identified as White, 6% as multi-racial, 4.4% as Black or African American, 1.1% as Asian, and 2.7% as other.

### 2.2. Measures

#### 2.2.1. Demographic Questionnaire

Participants were asked to provide general demographic information (i.e., age, gender identity, race, ethnicity, and year in school).

#### 2.2.2. Penn State Electronic Cigarette Dependence Index (PSECDI)

The PSECDI is a 10-item self-report questionnaire used to assess degree of e-cigarette dependence [15]. Participants were asked to provide information on the strength of urges to use, waking at night to use, the frequency of use, difficulty quitting, and the experience of cravings and withdrawal symptoms. Previous work supports the total score as a valid and reliable index of e-cigarette dependence [15], with higher scores indicating greater dependence.

#### 2.2.3. E-Cigarette Knowledge

Participants were asked investigator-generated questions to assess their knowledge of health risks associated with e-cigarette use and e-cigarette ingredients as well as where to go with questions related to e-cigarettes. Knowledge of health risks was assessed by asking “What are some of the negative health effects of using e-cigarettes? Check all that apply”. This prompt was accompanied by the following response options: “increased risk for heart attack, stroke, and coronary artery disease”; “increased risk for seizures”; “increased risk for depression”; “increased risk for lung diseases”; and “there aren’t any significant negative health effects”. These response options were chosen to reflect current health risks research, representing both well-publicized and lesser-known findings. Knowledge of e-cigarette ingredients was assessed by asking “Which of the following are in e-cigarettes? Check all that apply”. This prompt was accompanied by the following response options: “formaldehyde”; “volatile organic compounds”; “heavy metals (e.g., tin, lead, nickel, cadmium)”; “benzoic acid”; and “propylene glycol”. Finally, participants were asked “If you had questions about e-cigarettes, who would you be most likely to ask” and then chose one from the following response options: “a friend”; “someone who works in a vape shop”; “Google”; “doctor or other medical professional”; and “other”.

#### 2.2.4. E-Cigarette Use

Participants were asked investigator-generated questions related to the frequency of e-cigarette use in the past 30 days and whether or not they had ever changed the voltage on their device. Participants were also asked “How often do you use e-cigarettes and e-liquids as packaged vs. individualizing them”, followed by these response options: “use as packaged all of the time or almost all of the time”; “use as packaged most of the time”; “use as packaged about half the time”; “individualize most of the time”; and “individualize all or almost all of the time”.

### 2.3. Procedure

Data were collected between March 2020 and April 2021 as part of a larger survey on college student health. Undergraduate students at the University of Louisville who were aged 18 years or older were invited to participate in the study. Students were provided with a link to complete study measures online. Study data were collected and managed using REDCap (Research Electronic Data Capture) [16]. In order to ensure anonymity, information regarding participants’ IP addresses was not collected. Participants were granted course credit for their participation. The Institutional Review Board approved all study materials and procedures prior to data collection.

### 2.4. Data Analytic Plan

There were a total of 1642 survey responses. To ensure data quality, data were examined for correct responses to three quality-control questions that were randomly distributed throughout the survey (e.g., “*Choose option A for this question*”) and an affirmative answer to the question “*Have you carefully and accurately answered all the questions*” at the end of the survey. As a result, data from 568 participants were removed (*n* = 96 did not answer the quality-control questions correctly, *n* = 472 had missing data for the quality-control questions and nearly all other survey questions). An additional 891 participants were removed because they did not engage in e-cigarette use in the past 30 days (*n* = 868) or engaged in combustible cigarette use in the past 30 days (*n* = 23). Data analyses were completed on the remaining 183 participants using SPSS version 27. Prevalence rates were calculated for answers to each of the questions. Fisher’s exact test was used to determine whether answers to any of the questions differed by gender, race, or year in school.

## 3. Results

**Figure 1:**
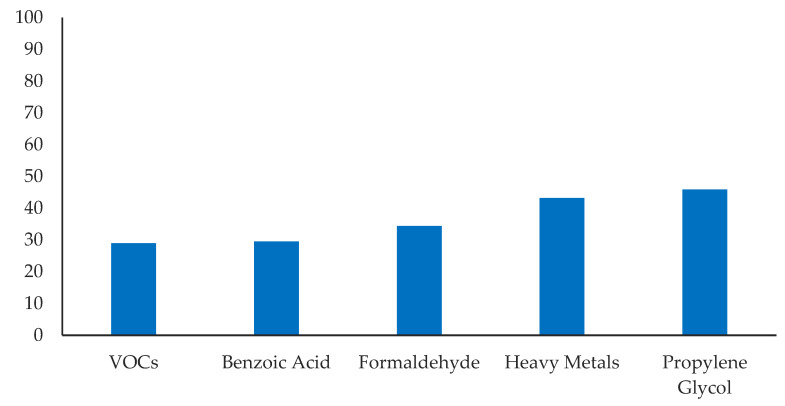
Percentage of participants who correctly recognized e-cigarette ingredients.

**Figure 2:**
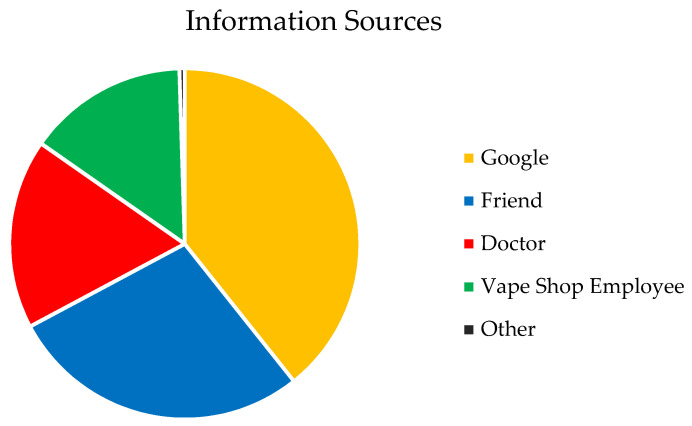
Percentage of participants who chose each source of e-cigarette information.

Participants reported using an e-cigarette on an average of 18.85 (*SD* = 11.25) days in the past 30 days, and 43.2% reported daily use. The average e-cigarette dependence score was 9.38 (*SD* = 5.10), indicating a moderate level of dependence. When asked about the negative health effects of e-cigarettes, most participants correctly recognized that e-cigarettes increased the risk of heart attack, stroke, and coronary artery disease (77.0%) and lung disease (89.1%). However, fewer than half of participants recognized the increased risk of seizures (43.7%) and depression (47%). Only one-third to one-half of participants correctly identified volatile organic compounds (29.0%), benzoic acid (29.5%), formaldehyde (34.4%), heavy metals (43.2%), and propylene glycol (45.9%) as ingredients in e-cigarettes (Figure 1). When asked about making modifications to their e-cigarette devices, 6.6% had changed the voltage at least once, and 21% individualized their e-cigarettes most to all of the time. When asked where they would go with questions about e-cigarettes, 39.3% said Google, 27.9% a friend, 17.5% a doctor or other medical professional, 14.8% a vape shop employee, and 0.5% other (Figure 2). There were no differences in prevalence rates for any of these answers based on gender, race, or year in school.

## 4. Discussion

The current study sought to examine college student e-cigarette users’ knowledge about e-cigarette ingredients and health risks, how often they make changes to their devices that might exacerbate risks, and whether they utilize reputable sources when searching for information regarding e-cigarettes. Overall, college student users are aware of the health risks that have received the most media attention (i.e., cardiovascular diseases and respiratory diseases). The proportion of participants in the current sample who were aware of these health risks is approximately 2.5 to 3 times higher than what was found in adult e-cigarette users [12]. It is possible that there are true differences between college students and adults; however, it may be that knowledge about health risks has increased in the time since the previous study was conducted. Further research is needed to better ascertain whether these increases have occurred across all age groups.

Participants were less familiar with the health risks associated with e-cigarette use that are not as widely discussed (i.e., seizures and depression). Not being aware of the association between e-cigarettes and depression, in particular, may place college student e-cigarette users at higher risk of depression. Moreover, due to the high rates of depression among college students [17,18], it is possible that e-cigarettes are being used as an affect regulatory strategy, similar to what is seen with combustible cigarettes [19,20,21]. Although college student e-cigarette users should be among the most informed of their age group, over half of participants were unaware of e-cigarette ingredients with significant potential for negative health impacts. This finding mirrors what has been reported in other work [13]. Unfortunately, this lack of knowledge may contribute to the perception that e-cigarettes are safe to consume.

It also appears that college student e-cigarette users are engaging in potentially risky practices. A substantial minority of e-cigarette users reported regularly making modifications to their devices, including changing the voltage, which can result in greater nicotine consumption and increased risk of exposure to carcinogens (e.g., formaldehyde) [22,23]. Further, very few participants would seek information about e-cigarettes from sources with the greatest likelihood of providing accurate information (i.e., a health care professional). Indeed, similar to findings in previous qualitative research [22,23], the majority of participants reported that they seek information from Google or their friends. This strategy is problematic as there is no quality control for the majority of web-based information. Further, individuals typically do not pay attention to the source of the information or consider its reliability, and few report going to health-related government sites (e.g., CDC) [24,25]. An examination of YouTube vaping-related content found that 85% of videos were produced by e-cigarette marketers, and 94% conveyed positive messages about vaping [26]. Thus, it is likely that college students are not receiving accurate information from the sources they are most likely to use.

These findings should be considered in the context of the study’s limitations. First, the sample consisted primarily of White females. Future research would benefit from using more diverse samples. Second, the questions about device modifications did not ask participants to specify what types of changes they were making. Thus, a clearer understanding of specific changes (e.g., relatively small to more major ones) would be helpful. Similarly, participants were not asked how often they sought information about e-cigarettes. Such information would help determine whether users who have more questions use certain resources more often. Finally, the current cross-sectional study did not allow for an examination of how knowledge and behavior patterns change over time with longer use.

## 5. Conclusions

The current findings suggest that college student e-cigarette users are relatively well-informed about many, but not all, health risks associated with e-cigarettes. However, they are relatively unaware of the harmful substances in e-cigarettes and seek information from less reliable sources. Targeted public health campaigns educating college students about e-cigarettes, particularly the risk for depression, and where to seek reliable, fact-based information about e-cigarettes, are needed.

## Data Availability

Data are available from the corresponding author upon reasonable request.
